# Anticancer Potential of Thieno[2,3-d]pyrimidine Derivatives in Oral Carcinoma Models

**DOI:** 10.3390/molecules31030397

**Published:** 2026-01-23

**Authors:** Ivan Iliev, Aleksandrina Nesheva, Anelia Mavrova, Denitsa Yancheva, Aneliya Kostadinova, Severina Semkova, Albena Momchilova, Iana Tsoneva, Galya Staneva, Biliana Nikolova

**Affiliations:** 1 Institute of Experimental Morphology, Pathology and Anthropology with Museum, Bulgarian Academy of Sciences, Acad. G. Bonchev Str., Bl. 25, 1113 Sofia, Bulgaria; taparsky@abv.bg; 2 Institute of Biophysics and Biomedical Engineering, Bulgarian Academy of Sciences, Acad. G. Bonchev Str., Bl. 21, 1113 Sofia, Bulgaria; nescheva@gmail.com (A.N.); aneliakk@yahoo.com (A.K.); severina.yordanova@gmail.com (S.S.); albena_momchilova@abv.bg (A.M.); itsoneva@bio21.bas.bg (I.T.); gstaneva@bio21.bas.bg (G.S.); 3 Department of Organic Chemistry, Faculty of Chemical Technologies, University of Chemical Technology and Metallurgy, S8 Kliment Ohridski Blvd., 1756 Sofia, Bulgaria; anmav@abv.bg; 4 Institute of Organic Chemistry with Centre of Phytochemistry, Bulgarian Academy of Sciences, Acad. G. Bonchev Str., Bl. 9, 1113 Sofia, Bulgaria; denitsa.pantaleeva@orgchm.bas.bg

**Keywords:** thienopyrimidines, oral squamous cell carcinoma, antiproliferative activity, apoptosis, cell cycle arrest, cytoskeleton, drug-likeness

## Abstract

Oral squamous cell carcinoma (OSCC) remains a major therapeutic challenge due to aggressive progression, high recurrence, and limited selectivity of current treatments. In this study, a series of seven 4-amino-2-substituted tetrahydrobenzothieno[2,3-d]pyrimidines were evaluated for their cytotoxic, antiproliferative, and mechanistic effects against oral cancer cell lines with different metastatic potential (HSC-3 and SCC-9), alongside non-tumorigenic keratinocytes (HaCaTs). Several compounds demonstrated selective anticancer activity, with Compounds **5** and **6** showing the most favorable balance between potency and selectivity. Antiproliferative assays revealed effective inhibition of cancer cell growth, while clonogenic assays confirmed a pronounced reduction in long-term survival, particularly in highly metastatic HSC-3 cells. Mechanistic studies indicated that the anticancer effects are associated with S-phase cell cycle arrest, apoptosis induction, and profound disruption of the actin cytoskeleton. In silico ADME and drug-likeness analyses supported the lead-like properties of the most active derivatives. Overall, these findings identify thienopyrimidine derivatives as promising scaffolds for the development of targeted therapies against OSCC and warrant further optimization and in vivo evaluation.

## 1. Introduction

Oral cancer is a malignant neoplastic formation on the mouth and the back of the throat. Oral cancers develop on the tongue, on the tissue lining the mouth and gums, under the tongue, at the base of the tongue, and the area of the throat at the back of the mouth.

According to the latest data from World Cancer Research Fund, 389,846 new cases of mouth and oral cancer were recorded in 2022, and this fact classified mouth and oral cancer as the 16th most common cancer worldwide [1]. It is the 12th most common cancer in men and the 18th most common cancer in women [1].

Oral cancer most often occurs in people over the age of 40 and affects more than twice as many men as women. Most cancers in the mouth are related to tobacco use, drinking alcohol, or both, and most throat cancers are caused by the human papilloma virus (HPV) [2,3].

The majority of oral cancers over 90% are defined as a squamous cell carcinoma (OSCC) with different levels of differentiations and possibility for regional lymph node metastasis [4,5].

Oral cancer is characterized by aggressive tumor progression, high recurrence rates, and poor patient survival. Despite advancements in conventional therapies, including surgery, radiation, and chemotherapy, treatment resistance and severe side effects remain significant challenges. Therefore, there is a growing interest in developing novel, targeted therapeutic agents with enhanced efficacy and reduced toxicity [6].

OSCC usually carries the epidermal growth factor (EGF) receptors. Tyrosine kinases (TK) are chemical messengers (enzymes) used by cells to control how they grow and divide. They possess on–off mode of action. After attachment of the growth factor to the cell receptor it switches the TK on. This signals cell to divide [7].

Tyrosine kinase inhibitors (TKIs) are a class of targeted therapy drugs that block the action of tyrosine kinases, enzymes involved in cell signaling pathways that regulate critical processes such as growth, differentiation, and survival. TKIs are used primarily in the treatment of various cancers and certain inflammatory and autoimmune diseases [8]. TKIs function by inhibiting the phosphorylation activity of tyrosine kinases, thereby preventing the activation of downstream signaling pathways that promote uncontrolled cell proliferation and survival. TKIs can be classified into two major categories: Receptor Tyrosine Kinase Inhibitors (RTKIs), which inhibit signaling pathways initiated by growth factor receptors such as EGFR, VEGFR, and HER2 [9], and Non-Receptor Tyrosine Kinase Inhibitors (NRTKIs) which target intracellular tyrosine kinases such as BCR-ABL and JAK kinases.

Overexpression of vascular endothelial growth factor (VEGF) was found in patients with head and neck squamous cell carcinoma [10].

Recently, salivary biomarkers for various cancers have become a subject of strong research interest. Several potential salivary biomarkers for early diagnosis of OSCC have been reported in the literature, including basic fibroblast growth factor (bFGF, FGF-2) [11]. Thienopyrimidine derivatives, a class of heterocyclic compounds containing a fused thiophene-pyrimidine scaffold, have demonstrated promising anticancer properties against various malignancies, including oral cancer [12].

Thienopyrimidines serve as structural analogs to TKIs. They consist of a fused thiophene and pyrimidine ring system, which allows them to interact with tyrosine kinase domains in a manner similar to traditional TKIs. These compounds have shown potential in targeting various kinases, including EGFR, VEGFR, and BCR-ABL [13].

Recent studies have shown that thienopyrimidine exerts their cytotoxic effects on oral cancer cells through multiple mechanisms, including induction of apoptosis, cell cycle arrest, inhibition of angiogenesis, and suppression of key oncogenic pathways such as PI3K/Akt, MAPK/ERK, NF-κB, and p53. Structural modifications of these compounds have further improved their anticancer potency, selectivity, and bioavailability. Notably, certain substituted thienopyrimidines act as tyrosine kinase inhibitors (TKIs), targeting epidermal growth factor receptors (EGFRs), the latter being often overexpressed in oral squamous cell carcinoma (OSCC). Some derivatives also function as DNA intercalators, disrupting cancer cell proliferation at the genetic level [14,15,16,17,18,19].

**Figure 1:**
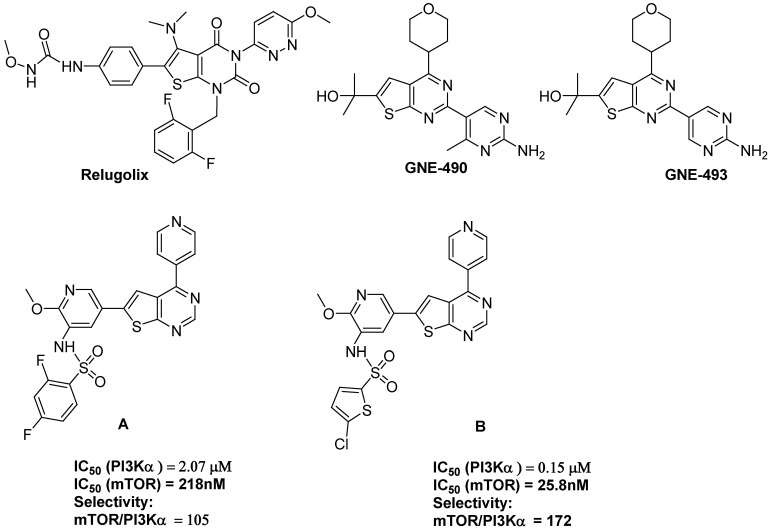
Thieno[2,3-d]pyrimidine drugs and TKIs A and B.

Figure 1 illustrates both anticancer thienopyrinidines drugs and tyrosine kinase inhibitors.

Relugolix (TAK-385) is a thienopyrimidine derivative that has successfully completed phase III of clinical trials. It is being investigated for its ability to treat endometriosis and prostate cancer by acting as a gonadotropin-releasing hormone receptor (GnRHR) antagonist [19]. GNE-493, a dual inhibitor of PI3K and mTOR from MedChemExpress NJ, USA, has been extensively studied for its effects on various cancer types. GNE-490 is a potent pan-PI3K inhibitor with IC_50_ values of 3.5 nM, 25 nM, 5.2 nM, and 15 nM for PI3Kα, PI3Kβ, PI3Kδ, and PI3Kγ, respectively. GNE-490 has >200-fold selectivity for mTOR (IC_50_ = 750 nM). GNE-490 shows potent suppression efficacy profile against MCF7.1 breast cancer xenograft model [14].

Furthermore, structure-activity relationship (SAR) studies have revealed that specific substitutions at the C2, C4, and C6 positions of the thienopyrimidine core significantly enhance anticancer activity. For example, halogenated derivatives (fluorine or chlorine substitutions) have been reported to increase cytotoxicity by improving cellular uptake and interaction with oncogenic proteins. In addition, thienopyrimidine hybrids conjugated with quinolines, indoles, or sulfonamides have exhibited enhanced selectivity toward cancer cells while minimizing toxicity to normal tissues.

Several recent investigations have also explored the potential of thienopyrimidine derivatives in combination therapy [15], showing that these compounds can sensitize oral cancer cells to standard chemotherapeutic agents like cisplatin and paclitaxel, overcoming drug resistance and enhancing overall treatment efficacy. Despite these promising results, challenges remain in terms of pharmacokinetics, solubility, and in vivo toxicity, necessitating further research and optimization [20].

Our studies have recently contributed significantly to the study of thienopyrimidine derivatives, especially focusing on their biological activity.

Despite advances in research and therapy, the survival rate of patients diagnosed with oral cancer has not improved significantly, which is why the challenge facing biomedical sciences is still open. By synthesizing new substituted thienopyrimidines we presume that we can register higher cytotoxicity and reduced side effects and thus improve the prognosis for treatment of diseases related to oral cancer.

The repurposing of the study of already known compounds or drugs for other bioactivities or on other cell lines is one of the important approaches in the strategy of development of new drugs. Therefore, we decided to investigate a series of thienopyrimidines synthesized earlier by us and to prove their activity against HCS-3 and SCC-9 cell lines. With this study, we aim to test whether the synthesized 4-amino-thienopyrimidines possess the desired anticancer properties against two different by their malignant potential and one normal oral cancer cell lines.

This research provides a comprehensive analysis of the impact of thienopyrimidine derivatives on oral cancer cell viability, focusing on their chemical structures, SAR insights, and therapeutic potential. Additionally, toxicity profiles, solubility, absorption, and pharmacokinetics are discussed. A deeper understanding of thienopyrimidine-based compounds could contribute to the development of novel targeted therapies, offering improved treatment options for oral cancer patients.

## 2. Results and Discussion

### 2.1. Biological Study

#### 2.1.1. Cytotoxicity Test

**Figure 2:**
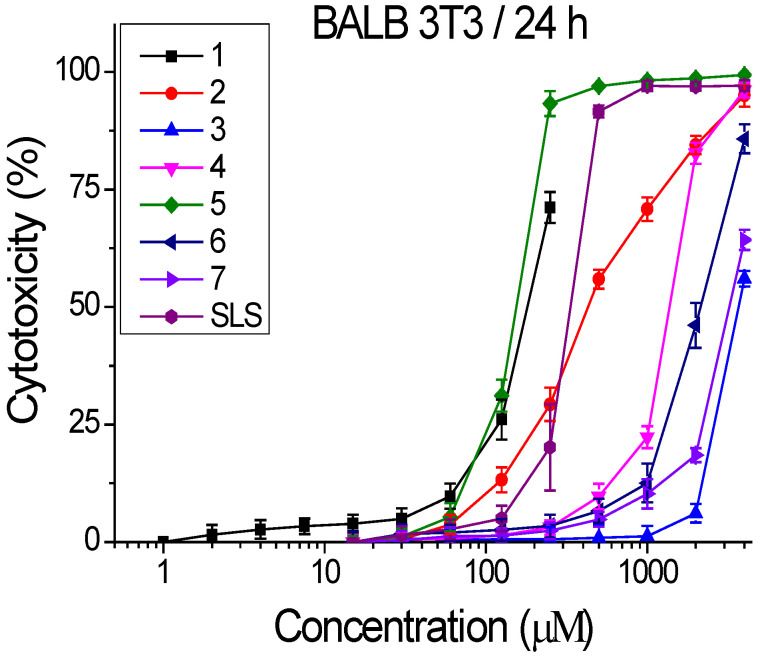
Dose–response curves for cytotoxicity of compounds **1**–**7**, determined in BALB3T3 cells. Values are means ± SD from three independent experiments: n = 6.

Compounds **1**–**7** were tested for cytotoxicity by an in vitro 3T3 NRU test, as described above. In this study, as a positive control SLS (sodium lauril sulfate) was used 1–250 µM. The concentrations of the tested compounds were in wide range from 15 to 4000 µM. The cells were cultivated with the compounds for 24 h, at 37 °C, 5% CO_2_, and 95% humidity. The cytotoxicity was expressed in percentages relative to the negative control. The effect was dose–response for all tested compounds. Results are shown in Figure 2.

Based on the data presented in Figure 2, the CC_50_ values (50% cytotoxic concentration) were calculated by nonlinear regression analysis (Table 1).

According to the results presented in Table 1, compounds **1**, **5**, and **2** possessed the highest cytotoxicity against BALB3T3 cells. Their levels of cytotoxicity are similar with those of the positive control—SLS. In contrast, compounds **3**, **6**, and **7** expressed the lowest cytotoxicity. The next step of our study was to evaluate the potential antiproliferative and anticancer effect of the compounds.

#### 2.1.2. Antiproliferative Activity

All studied compounds from **1** to **7** show lower levels of antiproliferative activity against control non-tumorigenic cell line HaCaT in contrast to the effect achieved in both cancer cell lines high metastatic HSC-3 and low metastatic SCC-9 potential (Table 2). Since the selectivity of anticancer drugs plays a key role in future treatment of oncological diseases, the index (SI) of the compounds was calculated and is also shown in Table 2.

Based on the data presented in Table 2, the MTT assay showed that doxorubicin, the positive control in this experiment, was extremely potent against both HSC-3 and SCC-9 cell lines (IC_50_ ≈ 0.18–0.40 μM). However, it exhibited low selectivity (SI = 1.55–3.64), indicating that it affects normal cells nearly as effectively as cancer cells. All test compounds possessed much higher IC_50_ values (lower potency) compared to doxorubicin but differed in selectivity.

Compound **1** was the most potent against both low-metastatic and high-metastatic cell lines (IC_50_ = 38 μM for SCC-9 and 65.7 μM for HSC-3), exhibiting the strongest cytotoxic activity among the tested compounds. Compound **5** showed the next highest activity (IC_50_ = 48.5 μM for SCC-9 and 58.9 μM for HSC-3), with potency comparable to that of Compound **1**. In contrast, Compounds **3**, **4**, and **7** were less potent, with IC_50_ values > 200 μM, indicating low cytotoxic activity.

Selectivity was calculated as the ratio of IC_50_ values for HaCaT cells to those for cancer cells, where higher SI values indicated greater selectivity toward cancer cell lines. Against the low-metastatic SCC-9 cell line, the highest SI was observed for Compound **6** (SI = 22.32), indicating strong selectivity despite its modest potency (IC_50_ = 67 μM). This was followed by Compound **4** (SI = 12.8), which was selective but less potent (IC_50_ = 207 μM), and Compound **3** (SI = 9.38), which also showed selectivity but weak potency (IC_50_ = 256 μM).

For the HSC-3 cell line, Compounds **6** (SI = 6.41) and **5** (SI = 3.0) exhibited the highest selectivity. Based on these results, Compounds **6** and **5** were selected for further studies against the HSC-3 cell line, while Compounds **6** and **4** were selected for SCC-9.

The lack of a strict correlation between cytotoxicity in non-tumor fibroblasts and oral cancer cells suggests that this does not directly predict the anticancer activity of the tested compounds in HSC-3 and SCC-9 cells. In this study, tumor selectivity was prioritized over absolute cytotoxic potency for lead identification. While some compounds (e.g., Compound **1**) exhibited strong anticancer activity, their lower selectivity indices suggest a narrower therapeutic window. In contrast, compounds such as **5** and **6** demonstrated a more favorable balance between anticancer activity and reduced cytotoxicity toward non-tumor fibroblasts, supporting their relevance as lead scaffolds.

In particular, the high selectivity index observed for Compound **6**, despite its moderate potency, is considered pharmacologically meaningful, as selectivity provides a robust foundation for subsequent lead optimization aimed at enhancing potency while preserving tumor specificity.

To broaden our investigation, we intentionally included compounds with differing cytotoxic profiles: one compound with low cytotoxicity common to both high- and low-metastatic oral cancer cell lines (Compound **6**), one compound with low cytotoxicity specific to SCC-9, and one compound with high cytotoxicity specific to HSC-3.

#### 2.1.3. Colony Forming Assay

The subsequent phase of the study involved a colony formation assay. This approach assesses the impact of long-term treatment on colony development following 9 days of exposure to IC_50_ concentrations of Compounds **5** and **6** in case of HSC-3 cell line and Compounds **4** and **6** against SCC-9. Evaluation of the capacity of individual cells to generate colonies (defined as clusters of at least 50 cells) provides an accurate estimation of the fraction of cells that maintain clonogenic potential. The colony formation assay is considered the gold standard to determine cellular sensitivity and to quantify the proportion of viable versus apoptotic cells under experimental conditions, applicable to both established cell lines and primary cell cultures.

**Figure 3:**
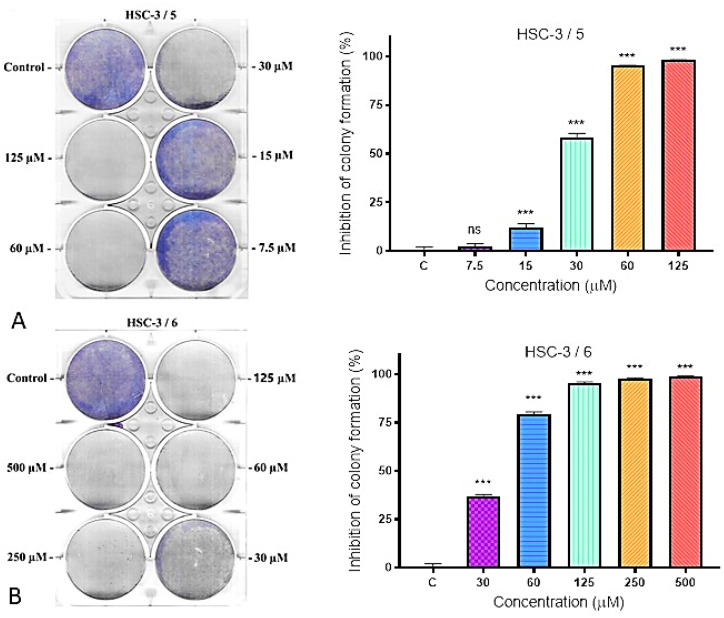
Inhibition of colony formation of Compounds **5** (**A**) and **6** (**B**) in HSC-3 cell line. Left coloring with MTT dye, right—graphical representation of the results in % inhibition of colony formation compared to the negative control (untreated cells).

**Figure 4:**
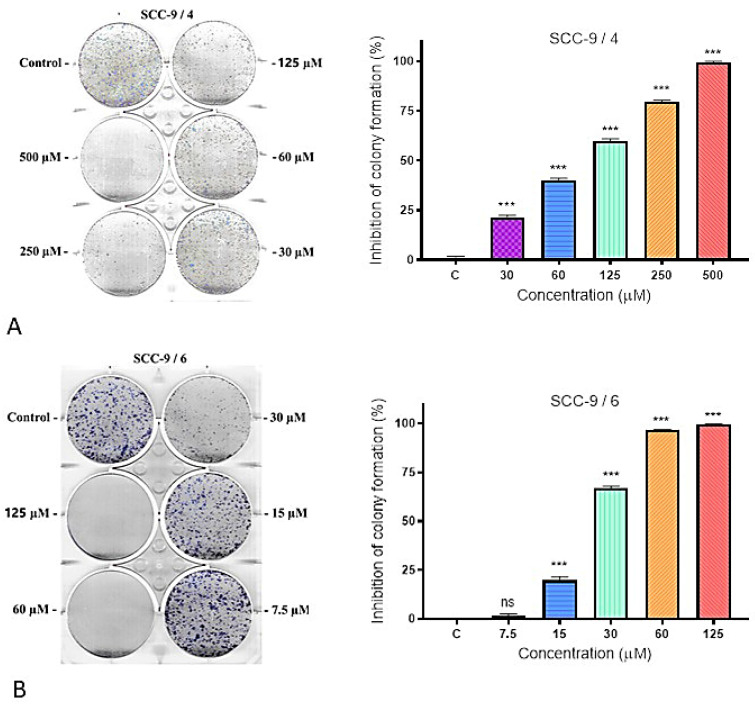
Inhibition of colony formation of Compounds **4** (**A**) and **6** (**B**) in SCC-9 cell line. Left coloring with MTT dye, right—graphical presentation of the results in % inhibition of colony formation compared to the negative control (untreated cells).

A strong anticlonogenic effect was observed after treatment of high metastatic cell line HSC-3 with different concentrations of Compound **5** (IC_50_ 26.89 µM). Even doses like IC_50_ induced more than 50% inhibition of colony formation and no colonies were found 9 days after treatment with concentrations of 60 and 125 µM compared to untreated control cells (Figure 3A). Less potent inhibition efficiency of colony formation was displayed by Compound **6** (IC_50_ 37.10 µM) (Figure 4B). The tested Compound **5** markedly inhibited colony formation in HSC-3, indicating a loss of clonogenic potential and suggesting strong cytotoxic and/or anti-proliferative activity.

The effect of treatment of the low-metastatic SCC-3 cell line with Compounds **4** and **6** is shown in Figure 4. The studied compounds exhibited a weak anticlonogenic effect. Even at the IC_50_ concentration (89.36 μM), Compound **4** did not induce 50% inhibition of colony formation. Compound **6** was even less effective.

The effect of treatment of both cell lines with the selected compounds can be defined as statistically significant reduction in clonogenic survival in a dose-dependent manner. These findings indicate that the tested compounds exert potent cytotoxic and/or long-term antiproliferative effects, leading to irreversible loss of clonogenic potential in oral cancer cells.

#### 2.1.4. Apoptosis and Cell Cycle Analysis by PI Staining

**Figure 5:**
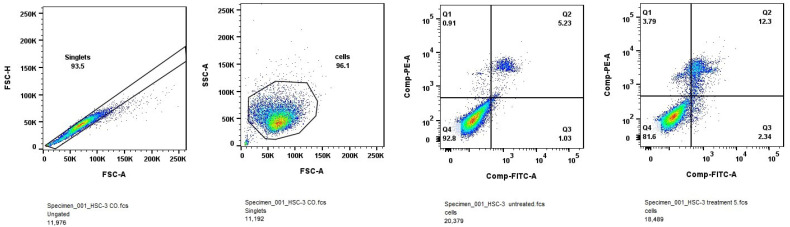
Apoptosis induction after 24 h treatment of HSC-3 cell line with IC_50_ of Compound **5**.

Subsequent studies included apoptosis assay and cell cycle assay. Evaluation of the effect of IC_50_ doses of the tested compounds on apoptosis induction and cell cycle arrest might help to elucidate the possible mechanism of action of thienopyrimidines. The results are presented in Figure 5.

After 24 h treatment of HSC-3 cell line with IC_50_ doses of Compound **5**, the increase in cells in early apoptosis was measured (1.03% in untreated control and 2.34% after treatment), but more obvious effect was observed in late apoptosis, where the increase was from 5.23% to 12.3%.

**Figure 6:**
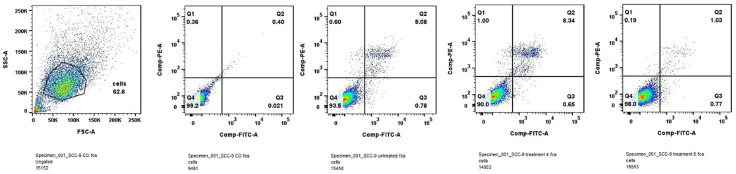
Apoptosis induction after 24 h treatment of SCC-9 cell line with IC_50_ of Compound **4** and Compound **6**.

SCC-9 cell line was treated with IC_50_ doses of Compound **4** and Compound **6** for 24 h. As shown in Figure 6, Compound **4** increased the number of cells in late apoptosis, while Compound **6** suppressed late apoptosis comparing with untreated control. These findings indicate that in SCC-9 cells Compound **6** may suppress proliferation through non-lethal or early stress-related mechanisms, rather than inducing extensive apoptotic cell death. Hallmarks of late apoptosis are as follows: membrane integrity is lost, DNA fragmentation occurs, and the cell is effectively dead Annexin V positive and PI/7-AAD positive.

A treatment-induced increase in late apoptotic cells after implies that the anticancer compound is effectively triggering programmed cell death, leading cells to reach the final phase of apoptosis—a desirable outcome, when testing potential anticancer agents. Compound **4** for SCC-9 cells was more effective in this way.

Next step of our study was to analyze the cell cycle after PI staining. Cell cycle arrest is a key therapeutic goal. Many anticancer agents including thienopirimidines induce cell cycle arrest to suppress tumor cell proliferation and trigger apoptosis. The data are shown in Table 3 and Table 4.

The phases G1, S, and G2 represent different stages of the cell cycle. According to the results presented in Table 3, most of the untreated cells (over 90%) were in G1 phase, meaning the cells are largely in a resting or growth phase. Very few cells were in the DNA synthesis (S) phase or preparing for mitosis (G2), indicating normal proliferation or a slow-dividing population. Cells treated with IC_50_ of Compound **6** were redistributed in cell cycle stages as follows: G1: 4.34%, S: 80.30%, and G2: 5.63%. A dramatic decrease in G1 and accumulation in S phase was observed. This suggests S-phase arrest, implying that Compound **6** interferes with DNA replication, preventing cells from progressing to G2 and mitosis. The small increase in G2 (5.63%) was negligible compared to S-phase accumulation. Compound **6** is likely to target DNA synthesis or the replication machinery, causing cytotoxicity primarily in cells actively replicating DNA.

After treatment with the IC_50_ concentration of Compound **5**, the cell-cycle distribution was as follows: G1: 18.20%; S: 73.20%; and G2: 0.88%. The G1 population decreased substantially, while the proportion of cells in the S phase increased, similar to the effect observed with Compound **6**. The G2 phase was almost completely depleted (0.88%), suggesting a strong arrest in the S phase, with very few cells progressing to G2.

In comparison with Compound **6**, both compounds induced S-phase arrest; however, Compound **5** appeared slightly more potent in reducing progression to the G2 phase, whereas Compound **6** resulted in a marginally higher accumulation of cells in the S phase.

Both showed a major disruption of the normal cell cycle, consistent with cytotoxic activity. Both Compound **5** and Compound **6** induced S-phase arrest, disrupting normal cell cycle progression. This suggests a mechanism of action that interferes with DNA replication.

Flow cytometric analysis of the cell cycle distribution revealed no substantial alterations in the proportions of cells in the G1, S, and G2 phases after treatment with IC_50_ concentrations of the tested compounds. In untreated SCC-9 cells, 50.60% were in the G1 phase, 29.30% in the S phase, and 13.20% in the G2 phase. Treatment with Compound **5** resulted in a nearly identical distribution (50.80% G1, 29.90% S, and 13.80% G2), indicating minimal effect on cell cycle progression. In contrast, Compound **6** induced a modest decrease in the S-phase population (26.40%) and a slight reduction in the G2 fraction (12.40%), suggesting a tendency toward G1 accumulation or potential cell cycle delay preceding DNA synthesis. Overall, none of the compounds produced a pronounced arrest at any specific cell cycle phase under the tested conditions.

#### 2.1.5. Actin Staining

To further deepen our study, we evaluated the effects of thienopyrimidine treatment on cytoskeletal integrity. Therefore, the IC_50_ concentrations determined from the antiproliferative activity assays were used for the tested compounds.

**Figure 7:**
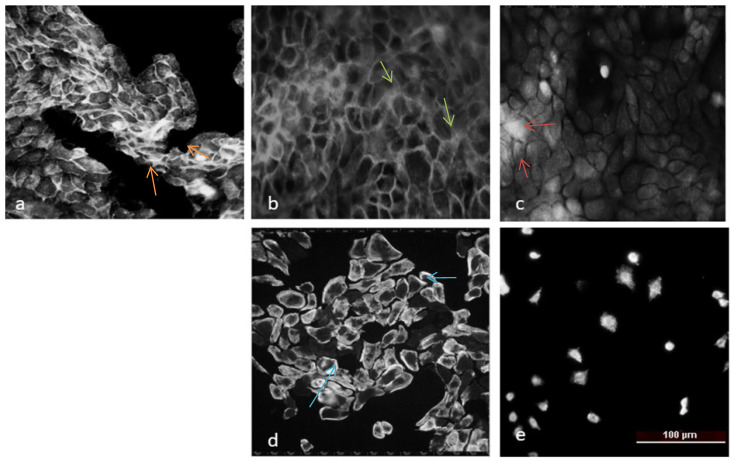
Actin staining of HaCaT cell line after treatment with IC_50_ concentration of studied Compounds: (**a**) untreated control, (**b**) Doxo—positive control, (**c**) compound **3**, (**d**) compound **4**, and (**e**) compound **6**. Arrows: orange—stress fibers, green—actin near to cell membrane, red—diffuse actin, and blue—actin in cells.

**Figure 8:**
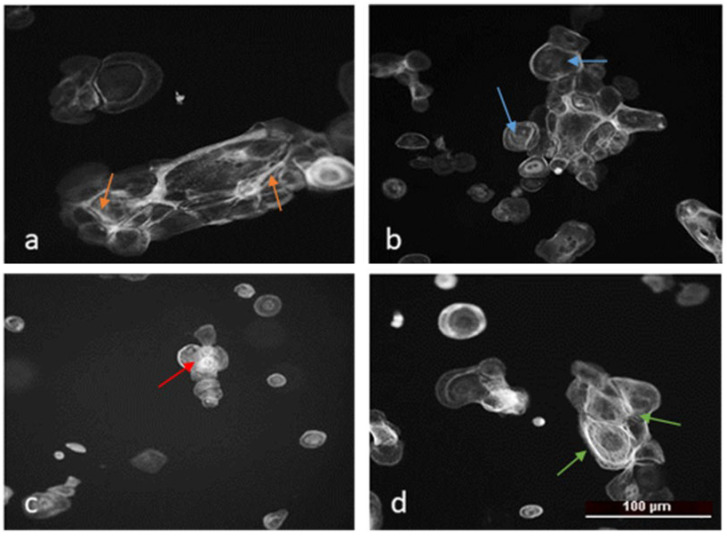
Actin staining of low metastatic SCC-9 cell line after treatment with IC_50_ concentration of studied compounds: (**a**) control, (**b**) Doxo—positive control, (**c**) Compound **4**, and (**d**) Compound **6**. Arrows: orange—stress fibers, blue—reorganization of actin in circle like, red—shrink cells, and green—peripheral organization of actin.

In control HaCaT cells (Figure 7a), phalloidin staining revealed a well-organized actin cytoskeleton characterized by well-defined stress fibers traversing the cytoplasm, as well as a distinct cortical actin ring. The cells displayed a flat, epithelial morphology and preserved intercellular contacts, consistent with strong adhesion and structural integrity of the cytoskeleton. After treatment with doxorubicin (Doxo)—positive control (Figure 7b), destabilization of the cytoskeleton was observed, which was manifested by loss of central stress fibers, increased peripheral accumulation of F-actin, and rounding of the cell body. These changes suggest a collapse of actin tension and possible initiation of apoptotic processes. Upon treatment with Compound **3** (Figure 8c), partial preservation of actin filaments was observed, together with a diffuse distribution of actin and an increase in intercellular spaces, consistent with disruption of cell–cell contacts. This suggests that Compound **3** induces enhanced cytoskeletal damage, whereas Compound **4** (Figure 7d) causes a marked peripheral condensation of actin and a reduction in filamentous structures. Some cells appeared shrunken and irregularly shaped, which is indicative of reduced cytoskeletal tension and eventual detachment from the substrate. The strongest effect was observed with compound **6** (Figure 7e), where cells showed a reduction in cell number, intense diffuse accumulation of F-actin, complete loss of stress fibers, and a highly rounded morphology. These findings suggest a profound cytoskeletal reorganization, likely associated with loss of adhesion and cell death.

An epithelial-like morphology was observed in Figure 8a in the untreated control of the low-metastatic SCC-9 cell line, although with greater variability in cell shape compared to HaCaT cells. The actin cytoskeleton contains fewer and less organized stress fibers, accompanied by diffuse peripheral actin signal, which is typical for transformed epithelial cells. After treatment with doxorubicin (b)—positive control, a pronounced disorganization of the cytoskeleton was observed as follows: F-actin was fragmented, stress fibers were absent, and peripheral signal was intensified, indicating cellular contraction and collapse of actin architecture. Following treatment with Compound **4** (d), cells became highly rounded, acquired a more compact shape, and F-actin was located mainly at the periphery, forming thin cortical structures, with weak internal filamentous signal. This phenotype may indicate significantly reduced cell adhesion and actin cytoskeleton reorganization. Finally, treatment with Compound **6** (e) resulted in dense but disorganized cortical actin accumulation, complete loss of stress fibers, and rounded cell morphology, consistent with cytoskeletal collapse and potential cell death.

**Figure 9:**
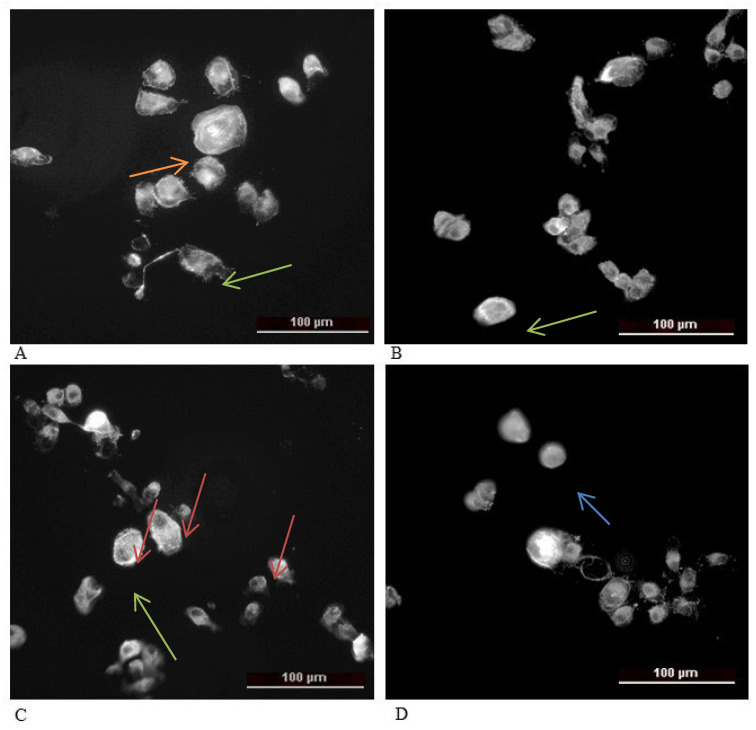
Actin staining of high metastatic HSC-3 cell line after treatment with IC_50_ concentration of studied compounds: (**A**) control, (**B**) Doxo—positive control, (**C**) Compound **5**, and (**D**) Compound **6**. Arrows: orange—stress fibers, green—circle like actin, red—actin near to the cell membrane, and blue—diffuse actine.

The actin cytoskeleton was analyzed by fluorescence microscopy images of HSC-3 cells (a highly metastatic human tongue squamous cell carcinoma line) following treatment with compound **5**, compound **6**, and doxorubicin, compared to untreated control cells (Figure 9). Quantitative evaluation of the peripheral-to-central actin ratio revealed distinct alterations in F-actin organization, reflecting changes in cell morphology, adhesion, and viability. In the present study, the cells were treated for 24 h at IC_50_ concentrations, a condition under which cytoskeletal disorganization and increased late apoptosis occur concurrently. Therefore, our data do not allow us to conclusively determine whether the disruption of F-actin organization represents a primary drug effect or a secondary consequence of apoptosis. The analysis of F-actin was used exclusively to monitor morphological changes and to qualitatively characterize the cellular response to treatment. Actin staining was not applied as a standalone marker of apoptosis or as evidence of a specific mechanism of action. Accordingly, the observed alterations in F-actin organization are interpreted as cytoskeletal changes accompanying the cytotoxic/apoptotic response, and the manuscript has been revised to avoid overinterpretation.

In control HSC-3 cells, the actin cytoskeleton displayed a characteristic polygonal morphology with well-developed stress fibers, clearly defined focal adhesions, and a peripheral actin ring, typical for strongly adherent and moderately motile epithelial tumor cells (Figure 9A).

Treatment with doxorubicin resulted in a complete disorganization of the actin network, pronounced cell rounding, and loss of peripheral actin structure. This phenotype is characteristic of cytotoxic damage, where cells lose adhesion and undergo apoptosis (Figure 9B).

Treatment with Compound **5** induced a partial disorganization of the actin network and a reduction in filament density, though the overall actin structure remained partially preserved. The cells appeared slightly rounded, suggesting weakened adhesion and a potential increase in susceptibility to cell death (Figure 9C).

Exposure to compound **6** caused a marked decrease in actin signal intensity and in the peripheral/central actin ratio. Fluorescence imaging revealed loss of stress fibers, diffuse cytoplasmic actin distribution, and a more rounded cell shape, indicative of significant cytoskeletal disassembly, reduced adhesion, and survival capacity (Figure 9D).

Overall, the actin cytoskeleton of control HSC-3 cells was well organized, consistent with their highly metastatic and migration-competent phenotype. Compound **5** induced moderate cytoskeletal destabilization associated with reduced adhesion, while compound **6** caused more profound disruption and loss of filamentous structures, suggesting a transition toward a weakly adherent, non-viable phenotype. Similarly, doxorubicin triggered complete cytoskeletal collapse and cytotoxicity.

We therefore refrain from interpreting the actin reorganization as evidence of migration inhibition. Instead, we describe it as a specific cytoskeletal alteration accompanying the cytotoxic/apoptotic response under the applied experimental conditions.

These findings indicate that the tested compounds strongly affect the actin dynamics of HSC-3 cells, leading to changes in cell adhesion, morphology, and viability. The diffuse and poorly organized actin filaments observed in the treated cells are more likely linked to cell death and decreased viability, rather than to enhanced migratory potential, which is otherwise a defining feature of this highly metastatic cell line.

The present study investigated the cytotoxic, antiproliferative and mechanistic effects of a novel series of thienopyrimidine derivatives (Compounds **1**–**7**) on normal and malignant oral epithelial cell lines, with a focus on understanding their potential as anticancer agents. The findings demonstrate that several of the synthesized compounds exerted selective cytotoxicity against oral squamous carcinoma cells (SCC-9 and HSC-3) while exhibiting reduced toxicity toward non-tumorigenic keratinocytes (HaCaTs). Furthermore, flow cytometric and fluorescence analyses suggest that these effects are associated with apoptosis induction, cell cycle arrest, and cytoskeletal disruption.

Based on the available experimental data, the compounds investigated in this study should be regarded primarily as cytotoxic/antiproliferative agents, rather than confirmed kinase inhibitors. Although the thieno[2,3-d]pyrimidine scaffold is commonly associated with kinase inhibition, no direct kinase inhibition assays were performed in the present study, and therefore a targeted kinase-based mechanism cannot be conclusively claimed.

Therefore, the compounds are considered as biologically active cytotoxic agents with potential for further mechanistic characterization.

##### Cytotoxicity and Antiproliferative Effects

The initial cytotoxic screening in BALB 3T3 fibroblasts revealed variable cytotoxicity among the tested compounds, with Compounds **1**, **2**, and **5** exhibiting the lowest CC_50_ values, comparable to the reference SLS. Despite their higher baseline cytotoxicity, these compounds were also among the most active against cancer cell lines, suggesting that structural features associated with increased membrane interaction or intracellular accumulation may contribute to their potency.

In contrast, Compounds **3**, **4**, **6**, and **7** displayed significantly higher CC_50_ values (>1300 µM), indicating lower general cytotoxicity. Interestingly, several of these less toxic compounds (notably Compound **6**) demonstrated marked selectivity toward cancer cells, reflected by high selectivity index (SI) values, particularly against the SCC-9 line (SI = 22.32). This implies that lower intrinsic cytotoxicity does not preclude anticancer potential; rather, selective targeting of malignant cells may occur through specific molecular interactions or stress responses unique to the tumor phenotype.

The antiproliferative assays confirmed that Compounds **1** and **5** were the most potent inhibitors of proliferation in both HSC-3 and SCC-9 cells, with IC_50_ values in the range of 38–65 µM. Compound **6**, though less potent, showed superior selectivity, suggesting differential mechanisms of action across the thienopyrimidine series. Such selectivity is particularly important in designing safer anticancer drugs, as conventional agents like doxorubicin displayed strong cytotoxicity but poor selectivity (SI < 4).

##### Clonogenic Survival and Long-Term Cytotoxicity

The colony formation assay provided additional insights into the long-term effects of treatment. Compounds **5** and **6** induced a pronounced reduction in the clonogenic potential of HSC-3 cells, with Compound **5** completely abolishing colony formation at concentrations above 60 µM. This irreversible loss of reproductive capacity indicates effective elimination of the most tumorigenic subpopulation—cancer stem-like cells capable of repopulation. In SCC-9 cells, the inhibitory effects of Compounds **4** and **6** were more modest, which may reflect differences in the metastatic potential or intrinsic resistance mechanisms between the cell lines. These findings are consistent with the observed selectivity patterns and suggest that the high-metastatic HSC-3 line is more vulnerable to thienopyrimidine-induced stress.

##### Apoptosis Induction

Flow cytometric analysis of Annexin V/PI staining demonstrated that treatment with Compound **5** (HSC-3) and Compound **4** (SCC-9) significantly increased the proportion of late apoptotic cells compared with untreated controls. The accumulation of Annexin V^+^/PI^+^ populations indicates progression to the terminal stages of apoptosis, involving loss of membrane integrity and nuclear fragmentation. Compound **6**, in contrast, exhibited a weaker pro-apoptotic effect, suggesting that its antiproliferative activity may arise primarily from cytostatic, rather than cytotoxic mechanisms. These results imply that apoptosis induction contributes, at least in part, to the anticancer activity of the more potent thienopyrimidines.

##### Cell Cycle Effects

Analysis of the cell cycle distribution revealed compound-dependent variations in arrest patterns. In HSC-3 cells, both Compounds **5** and **6** induced a pronounced accumulation of cells in the S phase, accompanied by a marked reduction in the G_1_ population. This redistribution indicates an interference with DNA replication, possibly through inhibition of replication fork progression or activation of intra-S checkpoint pathways (ATR/Chk1 signaling). The depletion of G_2_-phase cells further supports an S-phase arrest rather than a mitotic block. Such effects are characteristic of agents that disrupt nucleotide metabolism, DNA polymerase activity, or replication-associated repair mechanisms.

In contrast, SCC-9 cells treated with Compounds **4** and **6** displayed no major alterations in cell cycle phase distribution, with only a slight increase in G_1_-phase cells. This may indicate that these compounds exert their effects more through apoptosis or cytoskeletal disruption than through direct interference with cell cycle progression. Overall, the data suggest that thienopyrimidines can affect multiple regulatory pathways depending on the cellular context and compound structure.

##### Cytoskeletal Reorganization and Morphological Changes

Fluorescence staining of F-actin filaments revealed that thienopyrimidine treatment profoundly altered cytoskeletal architecture. In both normal (HaCaT) and cancer (SCC-9) cells, exposure to the active compounds, particularly Compound **6**, led to loss of stress fibers, cell rounding, and condensation of peripheral actin. These changes are hallmarks of cytoskeletal collapse and often precede detachment and apoptotic death. The disruption of actin organization may contribute to the observed decrease in clonogenic potential and support the hypothesis that thienopyrimidines impair cell adhesion and motility—key processes in cancer metastasis. The differential response between HaCaT and SCC-9 cells also emphasizes that malignant cells, with their inherently less organized cytoskeletons, are more susceptible to further actin disorganization. In the present study, actin staining was performed after 24 h treatment at IC_50_ concentrations, a condition under which cytoskeletal alterations occurred together with reduced clonogenic survival, S-phase arrest, and an increased proportion of late apoptotic cells. Under these conditions, the observed loss of stress fibers and cell rounding is more consistent with a decline in cell viability rather than with a selective impairment of migratory capacity.

##### Overall Mechanistic Implications

Taken together, the results suggest that thienopyrimidine derivatives exert their anticancer activity through a combination of S-phase cell cycle arrest, apoptosis induction, and cytoskeletal destabilization. Compounds **5** and **6**, in particular, appear to interfere with DNA replication and actin filament dynamics, leading to both cytostatic and cytotoxic outcomes. The observed selectivity toward oral carcinoma cell lines highlights their potential as major candidates for further optimization. Future studies should focus on identifying the precise molecular targets—such as CDKs, DPP-4, or other kinases—and on evaluating whether these compounds synergize with established chemotherapeutics or inhibit metastatic behavior in vivo.

##### Ligand Efficacy

A crucial objective in drug research is to gain a comprehensive understanding of the molecular properties that influence oral bioavailability, which is essential for designing effective new drug candidates. Two key factors in this process are lipophilicity and number of hydrogen bond donors. These properties tend to remain relatively stable in oral medications over time. The presence of hydrogen bond donors and acceptors affects the polar surface area of a molecule, which can hinder its ability to penetrate cell membranes. This aspect is closely linked to the molecule’s bioavailability. Therefore, it is vital to calculate lipophilicity and other physicochemical parameters for the compounds under investigation. These factors significantly impact their absorption, distribution, metabolism, excretion, and potential toxicity [21].

The topological polar surface area (TPSA) is an important metric for assessing the bioavailability of drug molecules. It is closely related to the hydrogen bonding potential of a compound. TPSA of studied compounds was noticed in the range of 25.87–153.50 Å [22].

Lipophilicity and the number of hydrogen bond donors appear to be key properties, as they remain essentially constant in oral drugs over time. That is why we researched the physicochemical properties of the compounds using SwissAdme software (https://www.swissadme.ch/) [23].

For a drug to be effectively absorbed by the body, it must be in a solution state in order to be absorbed. The solubility of a drug is essential to ensure that the right concentration enters the bloodstream, allowing the desired pharmacological response. Therefore, solubility is a key factor in determining the effectiveness of a new drug solution [24]. Accurate determination of drug solubility is crucial, especially in cases where solubility is poor, as this may indicate a need for methods to enhance it. As shown in Table S3, thienopyrimidine 5 is classified as soluble according to the three methods employed, including Log S (ESOL) [23,25,26]

Pharmacokinetics refers to the process that encompasses the following four key stages of a drug’s journey through the body: absorption, distribution, metabolism, and excretion (ADME). The transport of drugs across cell membranes is essential in pharmacokinetics.

The calculated results, given in the Supplementary section (Table S4), indicate that the tested thienopyrimidines could exhibit high gastrointestinal absorption. The derivatives cannot permeate through the BBB (blood–brain barrier) but they can bind to P-gp (P-Glycoprotein) substrate and consequently can be transported out of a cell [27]. The studied thienopyrimidine **1** may act as inhibitor of CYP (cytochrome P450 superfamily) enzymes, while compound **5** does not reveal inhibitory properties to 2C19, 2CD9 and 3A4, and compound **6** to 2CD9 and 3A4, respectively. The Log K_p_ value is a measure of the ability of a drug to penetrate the skin. The greater the negative value of Log K_p_, the less the drug’s ability to pass through the skin. This measurement is essential for determining potential dermal hazards and the extent of chemical absorption [28,29]. Compound **5** possesses a higher Log K_p_ in comparison to thienopyrimidines **1** and **6**.

The data related to drug-likeness pertains to five rules named after their authors. According to the calculated data compounds **1 **, **5**, and **6** meet the criteria for drug likeness (Table S5) [21,30,31,32,33].

Drug-likeness is an important concept in drug discovery that focuses on identifying both virtual and real molecules that fit within the chemical space considered drug-like. This classification is based on one or more physicochemical properties. As can be seen, the data obtained demonstrate that compounds **5** and **6** meet the criteria for lead-likeness and fall within the space mentioned above [34,35].

As evident from Figure 2, the chemical structure of the studied compounds consists of a tetrahydrobenzothieno[2,3-*d*]pyrimidine building block, as well as an amino group at the 4th position and various substituents at the second position of the ring. It is these differences in substituents at the second position that have a significant impact both on the cytotoxic activity and physic-chemical, respectively, pharmacokinetic properties of the compounds. Compound **1** revealed high anti-proliferative effect against HSC-3 and SCC-9 cell lines but its lipophilicity was higher than the lipophilicity of compounds **5** and **6**. Moreover, the selectivity index of thienopyrimidine 1 was significantly lower than the selective indices of **5** and **6**. The structure of compounds **5** and **6** contains diethyleneamine (morpholine ring) and chloroethylene fragments, characteristic of the structure of some anticancer agents (alkylating agents, gefitinib). It is known that such fragments can participate in interaction with DNA strands of cells by forming covalent bonds, preventing DNA replication. This finding corresponds to the results obtained from the apoptosis and cell cycle analysis.

## 3. Materials and Methods

### 3.1. Biological Assays

#### 3.1.1. Chemicals

The reference chemicals (phototoxic/non-phototoxic drugs) acridine orange (Loba chemie Ltd., Fishamend, Austria), was purchased from Sigma-Aldrich, Schnelldorf, Germany. The cell cultural reagents were Dulbecco’s Modified Eagle’s Medium (DMEM), DMEM:Ham’s F12, Eagle’s Minimal Essential Medium (EMEM), Fetal Bovine Serum (FBS), and antibiotics (penicillin and streptomycin). The disposable consumables were supplied by Orange Scientific, Brain-l’Allued, Belgium.

**Figure 10:**
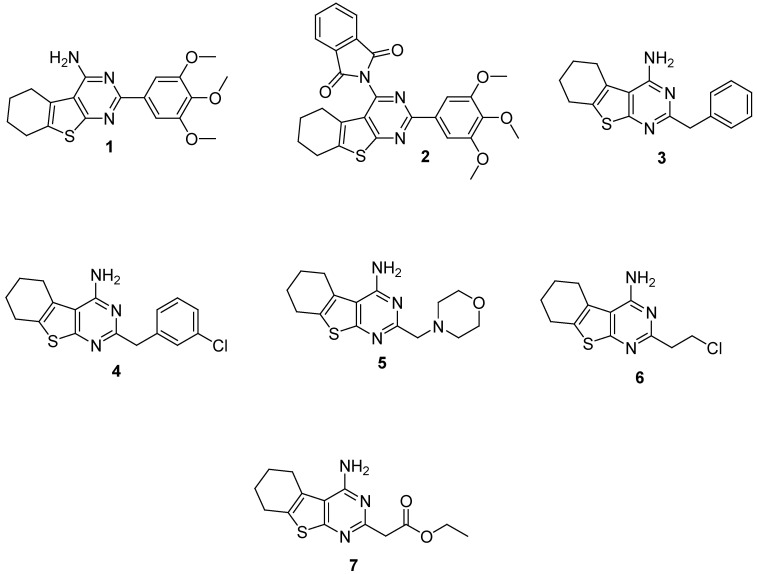
The compounds were synthesized according to previously reported procedures [36].

Compounds: 4-Amino-2-Substituted-Tetrahydrobenzothieno[2,3-d]pyrimidines 1–7 Figure 10.

#### 3.1.2. Cell Lines

All oral cancer cell lines (HaCaT, HSC-3, and SCC-9) were purchased from American Type Culture Collection-ATCC (Manassas, VA, USA). The HaCaT normal cell line was cultivated in DMEM, HSC-3 high metastatic cell line was cultivated in EMEM, and SCC-9 low metastatic cell line in DMEM:Ham’s F12 all media were supplemented with 10% fetal bovine serum (FBS), 1% sodium piruvate, and 1% MEM non-essential amino acids. All cell lines were maintained in a humidified atmosphere, with 5% CO_2_ at 37 °C.

#### 3.1.3. Cytotoxicity

BALB/3T3 cells were cultured in 75 cm^2^ tissue culture flasks in DMEM, 10% FBS, 2 mM glutamine, and antibiotics (penicillin 100 U/mL and streptomycin-100 μg/mL) at 37 °C, 5% CO_2_, and 90% relative humidity. Cytotoxicity was assessed by a validated BALB/3T3 clone A31 neutral red uptake assay (3T3 NRU test) [37]. Briefly, cells were plated in a 96-well microtiter plate at a density of 1 × 10^4^ cells/100 µL/well and incubated for 24 h. A wide concentration range was applied for the test compounds. After treatment with neutral red medium, washing, and treatment with the ethanol/acetic acid solution, the absorption was measured on a TECAN microplate reader (TECAN, Grödig, Austria) at a wavelength of 540 nm.

#### 3.1.4. In Vitro Anti-Proliferative Activity

Using the standard MTT-dye reduction assay described by Mosmann et al. [38], the anti-proliferative activity was tested. The method is based on the metabolism of the tetrazolium salt MTT to insoluble formazan. The formazan absorption was registered using a microplate reader at λ = 540 nm. The measured absorption is an indicator of cell viability and metabolic activity. Anti-proliferative activities were expressed as IC_50_ values (concentrations required for 50% inhibition of cell growth).

#### 3.1.5. Colony Forming Assay

The in vitro cell survival assay was performed with minor modifications according to Franken et al. [39]. In brief, HaCaT, HSC-3, and SCC-9 cells were seeded in six-well plates at a density of 500 cells/mL. The cells were allowed to adhere overnight and treated the next day with the selected concentration of the compounds. After 9 days of incubation, the medium was removed, cell colonies were stained with a 2% solution of methylene blue in 50% ethanol, and all colonies consisting of more than 50 cells were enumerated.

#### 3.1.6. Cell Cycle Analysis by Propidium Iodide (PI) Staining

The cells were trypsinized, suspended in medium + 10% FCS, and centrifuged (1000 rpm, 5 min); then, the pellet was suspended in PBS (1 mL) and fixed with EtOH. The cells were suspended in 2.5 mL pre-chilled 70% EtOH and incubated on ice for 15 min. The staining procedure was as follows: the pellet was suspended in a 500 µL PI-solution in PBS and 0.1 mg/mL RNase A, and 0.05% Triton X-100 were added. After incubation for 10 min at 37 °C, the cells were transferred to the flow cytometer and cell fluorescence was measured. The samples were measured by flow cytometry (LSR II, BD Biosciences, Franklin Lakes, NJ, USA) and analyzed using BD FACS DIVA software v. 6.1.3 (BD Biosciences).

#### 3.1.7. Actin Staining

After a 24 h incubation with the tested compounds, the cells were washed with phosphate-buffered saline (PBS; Gibco, Thermo Fisher Scientific, Waltham, MA, USA) and fixed with 3% paraformaldehyde (PFA; Electron Microscopy Sciences, Hatfield, PA, USA) in PBS for 15 min at room temperature. Following fixation, the samples were washed again with PBS and permeabilized with 0.5% Triton X-100 (Merck, Darmstadt, Germany) in PBS for 5 min. Non-specific binding sites were blocked by incubating the cells with 1% bovine serum albumin (BSA; Sigma-Aldrich, Schnelldorf, Germany) in PBS for 10 min. The cells were then incubated for 30 min at room temperature in the dark with Phalloidin–TRITC (Sigma-Aldrich, Schnelldorf, Germany; Cat. No. P1951), diluted 1:200 in PBS; after incubation, the samples were washed three times with PBS and once with distilled water. The coverslips were mounted onto glass microscope slides using Mowiol^®^ 4-88 (Sigma-Aldrich, Schnelldorf, Germany) as the mounting medium. Fluorescence imaging was performed using a Leica DMI 3000 B fluorescence microscope (Leica Microsystems GmbH, Wetzlar, Germany).

#### 3.1.8. Statistical Analysis

All experiments were performed in three independent biological replicates, each containing triplicate technical measurements, unless otherwise specified. Data are presented as mean ± standard deviation (SD). For cytotoxicity (CC_50_) and antiproliferative (IC_50_) assays, concentration–response curves were fitted using nonlinear regression in GraphPad Prism (version 9.5.1; GraphPad Software, San Diego, CA, USA). CC_50_ and IC_50_ values were calculated with 95% confidence intervals. Group comparisons were analyzed using one-way analysis of variance (ANOVA) followed by Bonferroni’s multiple-comparison test. For apoptosis and cell cycle data, statistical differences between treated and control samples were evaluated using either unpaired two-tailed Student’s *t*-tests (two groups) or one-way ANOVA (multiple groups), depending on the experimental design. A probability level of *p* < 0.05 was considered statistically significant for all analyses.

## 4. Conclusions

In this study, a series of thienopyrimidine derivatives (Compounds **1**–**7**) were evaluated for their cytotoxic, antiproliferative, and mechanistic effects on oral cancer cell lines (HSC-3 and SCC-9) and non-tumorigenic keratinocytes (HaCaTs). The results demonstrate that these compounds exhibit selective anticancer activity, with some derivatives (notably Compounds **5** and **6**) effectively reducing cancer cell proliferation while sparing normal cells.

Mechanistic investigations revealed that the anticancer effects involve S-phase cell cycle arrest, apoptosis induction, and actin cytoskeleton disruption, leading to impaired DNA replication, loss of clonogenic potential, and morphological changes consistent with programmed cell death, which is also confirmed by the results of the structure-activity relationship analysis. The degree of selectivity and potency varied among the compounds, highlighting the potential to optimize structural features for maximal therapeutic effect with minimal toxicity. The SAR assessment revealed that compounds **5** and **6** meet the criteria for lead- and drug-likeness. On the base of the combined cytotoxicity (IC50 58.96 μM on HCS-3 and IC50 48.54 ± 3.047 μM on SCC-9), selectivity 3 resp 3.6, clonogenic potential at concentration of 60 μM, interferation with DNA replication and actin filament dynamics, leading to both cytostatic and cytotoxic outcomes, ADME data (compound meets the criteria for lead-likeness), we considered the prioritization of compound **5** as primary lied.

Overall, thienopyrimidine derivatives represent a promising class of compounds for further development as targeted anticancer agents against oral squamous cell carcinoma. Future studies should focus on elucidating their precise molecular targets, evaluating their in vivo efficacy, and exploring potential synergistic combinations with existing chemotherapeutics.

## Figures and Tables

**Table 1:** Cytotoxicity of compounds **1**–**7** to BALB3T3 cells.

Compounds	Mean CC_50_ ± SD (μM)
**1**	180.70 ± 8.584
**2**	429.80 ± 20.354
**3**	3687.18 ± 72.436
**4**	1374.28 ± 22.543
**5**	154.44 ± 5.149
**6**	2130.59 ± 161.727
**7**	3222.59 ± 50.494
SLS *	314.23 ± 8.64

* SLS (Sodium lauryl sulfate)—positive control.

**Table 2:** Antiproliferative activity and selectivity index (SI) of compounds **1**–**7** and Doxorubicin (Doxo).

Compounds	Mean IC_50_ ± SD (μM)			SI	
	HaCaT	HSC-3	SCC-9	HSC-3	SCC-9
**1**	89.10 ± 5.42	65.76 ± 5.68	38.03 ± 1.89	1.36	2.35
**2**	203.17 ± 11.82	171.63 ± 2.91	67.15 ± 2.77	1.18	3.03
**3**	2398.74 ± 82.13	1356.62 ± 14.49	255.77 ± 20.25	1.77	9.38
**4**	2649.40 ± 99.82	1273.12 ± 48.09	206.97 ± 16.67	2.08	12.80
**5**	176.77 ± 1.10	58.96 ± 3.86	48.54 ± 3.047	3.00	3.64
**6**	1503.79 ± 102.70	234.58 ± 12.93	67.37 ± 5.89	6.41	22.32
**7**	2402.75 ± 26.98	929.14 ± 49.45	270.66 ± 13.85	2.59	8.88
**Doxo**	0.66 ± 0.03	0.43 ± 0.03	0.18 ± 0.02	1.55	3.64

Doxorubicin (Doxo)—positive control.

**Table 3:** Cell cycle analysis of HSC-3 cell line after treatment with IC_50_ concentrations of Compounds **5** and **6**.

Phases	Untreated	IC_50_ Compound 5	IC_50_ Compound 6
G1	91.60%	18.20%	4.34%
S	4.06%	73.20%	80.30%
G2	3.75%	0.88%	5.63%

**Table 4:** Cell cycle analysis of SCC-9 cell line after treatment with IC_50_ concentrations of Compound **4** and Compound **6**.

Phases	Untreated	IC_50_ Compound 5	IC_50_ Compound 6
G1	50.60%	50.80%	50.90%
S	29.30%	29.90%	26.40%
G2	13.20%	13.80%	12.40%

## Data Availability

The data are stored by the authors and, if there is a valid reason, they can be sent via personal correspondence.

## Supplementary Materials

The following supporting information can be downloaded at https://www.mdpi.com/article/10.3390/molecules31030397/s1, Table S1: Physicochemical Properties, Table S2: Lipophilicity of compound **1**, **5** and **6**, Table S3: Water solubility of the studied compounds 1, 4, 5, and 6, Table S4: Pharmacocinetic parameters of compound **1**, **5**, and **6**, Table S5: Drug likeness and Medicinal chemistry scores of compounds **1**, **4**, **5**, and **6**.

## Abbreviations

The following abbreviations are used in this manuscript:

OSCC	Oral Squamous Cell Carcinoma
HSC-3	Human Tongue Squamous Cell Carcinoma Cell Line (high metastatic)
SCC-9	Oral Squamous Cell Carcinoma Cell Line (low metastatic)
HaCaT	Human Keratinocyte Cell Line
ADME	Absorption, Distribution, Metabolism, and Excretion
HPV	Human Papilloma Virus
EGF	Epidermal Growth Factor
TK	Tyrosine Kinase
TKIs	Tyrosine Kinase Inhibitors
RTKIs	Receptor Tyrosine Kinase Inhibitors
EGFR	Epidermal Growth Factor Receptor
VEGFR	Vascular Endothelial Growth Factor Receptor
HER2	Human Epidermal Growth Factor Receptor 2
NRTKIs	Non-Receptor Tyrosine Kinase Inhibitors
BCR-ABL	Breakpoint Cluster Region–Abelson Kinase
JAK	Janus Kinase
VEGF	Vascular Endothelial Growth Factor
bFGF	Basic Fibroblast Growth Factor
FGF-2	Fibroblast Growth Factor-2
NF-κB	Nuclear Factor Kappa B
p53	Tumor Protein p53
GnRHR	Gonadotropin-Releasing Hormone Receptor
mTOR	Mammalian Target of Rapamycin
IC_50_	50% Inhibitory Concentration
SAR	Structure–Activity Relationship
NRU	Neutral Red Uptake
SLS	Sodium Lauryl Sulfate
CO_2_	Carbon Dioxide
SD	Standard Deviation
CC_50_	50% Cytotoxic Concentration
SI	Selectivity Index
MTT	3-(4,5-Dimethylthiazol-2-yl)-2,5-Diphenyltetrazolium Bromide
PI	Propidium Iodide
RNase	Ribonuclease
PBS	Phosphate-Buffered Saline
PFA	Paraformaldehyde
BSA	Bovine Serum Albumin
CYP	Cytochrome P450
BBB	Blood–Brain Barrier
P-gp	P-Glycoprotein
TPSA	Topological Polar Surface Area
ANOVA	Analysis of Variance
ATCC	American Type Culture Collection
