# Supplementary material for: Anticancer Potential of Thieno[2,3-d]pyrimidine Derivatives in Oral Carcinoma Models
Source: Molecules. 2026 Jan 23;31(3):397. doi: 10.3390/molecules31030397 (PMC12899554; doi:10.3390/molecules31030397)
Supplement: Supplementary file 1 [file molecules-31-00397-s001.zip › molecules-4080199-supplementary.pdf]

Supplementary Materials

"Anticancer Potential of Thieno[2,3-d]pyrimidine Derivatives in Oral Carcinoma Models"

"Biological Evaluation of 2-Substituted 4-Amino-Thieno[2,3-d]pyrimidines as Potential Anticancer Agents Against Oral Cancer Cell Lines in Oral Cancer Cell Lines with Different Malignancy"

Ivan Iliev[1], Aleksandrina Nesheva[2], Denitsa Yancheva[3], Anelia Mavrova[4], Aneliya Kostadinova[2], Severina Semkova[2], Albena Momchilova[2], Iana Tsoneva[2], Galya Staneva[2], Biliana Nikolova[2]

[1] Institute of Experimental Morphology, Pathology and Anthropology with Museum, Bulgarian Academy of Sciences, Acad. G. Bonchev Str., Bl. 25, Sofia, 1113, Bulgaria;
[2] Institute of Biophysics and Biomedical Engineering, Bulgarian Academy of Sciences, Acad. G. Bonchev Str., Bl. 21, Sofia, 1113, Bulgaria
[3] Institute of Organic Chemistry with Centre of Phytochemistry, Bulgarian Academy of Sciences, Acad. G. Bonchev Str., bl. 9, 1113 Sofia, Bulgaria
[4] Department of Organic Chemistry, Faculty of Chemical Technologies, University of Chemical Technology and Metallurgy, S8 Kliment Ohridski Blvd., 1756 Sofia, Bulgaria
* Correspondence:

Contents:

1. **Table S1. Physicochemical properties of compounds 1, 5, and 6 ……………..…p.2**
2. **Table S2. Lipophilicity parameters of compounds 1, 5, and 6 calculated by different models……………………………………………………………..…p.2**
3. **Table S3. Predicted water solubility of compounds 1, 4, 5, and 6 using different in silico methods…………………………………………………………..…p.2**
4. **Table S4. Predicted pharmacokinetic (ADME) parameters of compounds 1, 4, 5, and 6……………………………………………………………………..…p.3**
5. **Table S5. Drug-likeness and medicinal chemistry properties of compounds 1, 4, 5, and 6…………………………………………………………………….…p.3**

Table S1. Physicochemical Properties

Physicochemical Properties	Compound 1	Compound 5	Compound 6
Molecular weight (g/mol)	371.45	304.41 g/mol	267.78 g/mol
Num. heavy atoms	26	21	17
No. arom. heavy atoms	15	9	9
Fraction Csp3	0.37	0.60	0.50
No. rotatable bonds	4	2	2
No. H-bond acceptors	5	4	2
Num. H-bond donors	1	1	1
Molar Refractivity	104.16	89.23	73.82
TPSA	107.73 Å^2	92.51 Å^2	80.04 Å^2

Table S2. Lipophilicity of compound 1, 5 and 6

**Lipophilicity**	Compound 1	Compound 5	Compound 6
Log Po/w (iLOGP)	3.61	2.94	2.60
Log Po/w (XLOGP3)	4.01	1.93	3.32
Log Po/w (WLOGP)	3.85	1.46	2.94
Log Po/w (MLOGP)	2.13	1.42	2.58
Log Po/w (SILICOS-IT)	4.80	3.45	4.51
Consensus Log Po/w	3.68	2.24	3.19

Table S3. Water solubility of the studied compounds 1, 4, 5, and 6

Water solubility	Compound 1	Comp. 4	Compound 5	Compound 6
Log *S* (ESOL)	-4.83	-5.42	-3.85	
Solubility (mg/ml) (mol/l)	5.47e-03 1.47e-05	1.25e-03 3.80e-06	2.27e-01 7.44e-04	3.77e-02 1.41e-04
Class	Moderately soluble	Moderately soluble	Soluble	Soluble
Log *S* (Ali)	-5.97	-6.44	-3.50	-4.68
Solubility (mg/ml) (mol/l)	3.94e-04 1.06e-06	1.19e-04 3.62e-07	9.70e-02 3.19e-04	5.63e-03 2.10e-05
Class	Moderately soluble	Poorly soluble	Soluble	Moderately soluble

Log *S* (SILICOS-IT)	-6.19	-6.86	-3.92	-4.77
Solubility (mg/ml) (mol/l)	2.38e-04 6.41e-07	4.56e-05 1.38e-07	3.62e-02 1.19e-04	4.51e-03 1.68e-05
Class	Poorly soluble	Poorly soluble	Soluble	Moderately soluble

Table S4. Pharmacocinetic parameters of compound **1**, **5**, and **6**

**Pharmacokinetics**	**Compounds**			
	**1**	**4.**	**5**	**6**
GI absorption	High	High	High	High
BBB permeant	No	No	No	No
P-gp substrate	Yes	Yes	Yes	Yes
CYP1A2 inhibitor	Yes	Yes	Yes	Yes
CYP2C19 inhibitor	Yes	Yes	No	Yes
CYP2C9 inhibitor	Yes	Yes	No	Yes
CYP2D6 inhibitor	Yes	No	Yes	No
CYP3A4 inhibitor	Yes	Yes	No	No
Log K_p (skin permeation) cm/s	-5.72	-4.75	-6.79	-5.58

Table S5. Drug likeness and Medicinal chemistry scores of compounds 1, 4, 5, and 6

**Drug likeness**	**Compound 1**		**Compound 5**	**Compound 6**
Lipinski	Yes; 0 violation		Yes; 0 violation	Yes; 0 violation
Ghose	Yes		Yes	Yes
Veber	Yes		Yes	Yes
Egan	Yes		Yes	Yes
Muegge	Yes		Yes	Yes
Bioavailability Score	0.55		0.55	0.55
**Medicinal chemistry**	Compound 1	Compound 4	Compound 5	Compound 6
PAINS	0 alert		0 alert	0 alert
Brenk	0 alert		0 alert	1 alert: alkyl_halide
Leadlikeness	No; 2 violations: MW>350, XLOGP3>3.5		Yes	Yes
Synthetic accessibility	3.37		3.21	2.96
